# Magnitude and Clinical Predictors of Blood Pressure Changes in Patients Undergoing Hyperbaric Oxygen Therapy: A Retrospective Study

**DOI:** 10.3390/ijerph17207586

**Published:** 2020-10-19

**Authors:** Simone Schiavo, Carine Djaiani, Julian DeBacker, Lisa Albertini, Daniel Santa Mina, Stephanie Buryk-Iggers, Marcus Vinicius De Moraes, Mohammad Kanj, Rita Katznelson

**Affiliations:** 1 Hyperbaric Medicine Unit, Department of Anesthesiology and Pain Medicine, Toronto General Hospital, University Health Network, University of Toronto, Toronto, ON M5G 2C4, Canada; simone.schiavo@mail.utoronto.ca (S.S.); carine.djaiani@hotmail.com (C.D.); debacker.jw@gmail.com (J.D.); marcus.moraes@me.com (M.V.D.M.); mohammad.kanj@mail.mcgill.ca (M.K.); 2 Division of Cardiology, Department of Medicine, Toronto General Hospital, University Health Network, University of Toronto, Toronto, ON M5G 2C4, Canada; lisa.albertini@mail.utoronto.ca; 3 Faculty of Kinesiology and Physical Education, University of Toronto, Toronto, ON M5G 2C4, Canada; daniel.santamina@uhn.ca (D.S.M.); stephanie.buryk@uhnresearch.ca (S.B.-I.); 4 Department of Surgery, Faculty of Medicine, University of Toronto, Toronto, ON M5G 2C4, Canada; 5 Department of Supportive Care, Princess Margaret Cancer Centre, Toronto, ON M5G 2C4, Canada; 6 Department of Anesthesiology and Pain Management, University Health Network, Toronto General Hospital, University of Toronto, Toronto, ON M5G 2C4, Canada

**Keywords:** hyperbaric oxygen therapy, hypertension, blood pressure, hemodynamic change

## Abstract

Hyperbaric oxygen therapy (HBOT) is widely used to treat several pathologies. The hemodynamic changes during HBOT, particularly the magnitude of arterial blood pressure (ABP) increase, are not completely understood. No clinical predictors for HBOT-induced ABP increase have been described. The purpose of this study was to quantify ABP changes in patients undergoing HBOT and to examine their predictors. This retrospective longitudinal cohort study examined 3291 elective HBOT sessions. Non-invasive ABP was recorded before and after each session. The primary outcome was to quantify the HBOT-induced ABP rise. The secondary outcome was to determine the ABP-rise predictors among demographic and clinical variables. Overall, ABP increased significantly after HBOT; this finding was more evident in the hypertensive subgroup compared to the normotensive one (+6 vs. +16.2 mmHg). Clinical predictors of significant post-HBOT ABP change were history of hypertension and pre-session baseline ABP classification. This study demonstrates an absolute HBOT-induced ABP rise. This change is clinically relevant in patients with history of hypertension. A higher baseline ABP seems a risk factor for clinically relevant ABP change. Pre-session ABP should be used clinically as an indicator for strict ABP monitoring during HBOT; future studies are recommended to explore the ABP optimization before starting an HBO treatment.

## 1. Introduction

Hyperbaric oxygen therapy (HBOT) is an evidence-based elective intervention for a wide range of clinical indications as well as an emergency treatment for life-threatening conditions such as carbon monoxide poisoning, decompression sickness and arterial gas embolism [1]. Although, HBOT is generally considered extremely safe, there are a number of side effects that may include claustrophobia, middle ear barotrauma, vision changes, pulmonary oxygen toxicity, seizures, and cardiac dysfunction [2,3,4,5].

HBOT is associated with hemodynamic changes particularly pertinent to an increase in arterial blood pressure (ABP) and a decrease in heart rate [6]. However, the effects of HBOT on cardiovascular physiology are not completely understood [7]. A marked hyperoxic vasoconstriction is suggested to be a protective counterbalance response to the extreme hyperoxemia that is observed during HBOT [6]. The mechanism triggering vasoconstriction is related to the loss of vasorelaxation that is generated by hyperoxia-induced oxidation of nitric oxide radicals produced by the endothelium [6]. Additional contributors to vasoconstriction include a decrease of vasodilatory prostaglandins, an increase of vasoconstrictor endothelin-1 [8], and potentially a direct stimulation of the sympathetic nervous system producing an increase in plasmatic epinephrine and norepinephrine [7], even though the latter has been recently questioned suggesting a direct inhibition of the sympathetic outflow [9]. Overall, the arteriolar vasoconstriction leads to increased systemic vascular resistance causing arterial hypertension [6,10,11,12], which results in stimulation of baroreceptors [9] and cardio-inhibitory center [13,14] with subsequent increase in parasympathetic activity and decreased heart rate.

Despite these clinical findings, there are conflicting results about the incidence and magnitude of HBOT induced hemodynamic changes. Several studies have shown only mild fluctuations in ABP during HBOT [8,15,16], while one study showed a significant increase in ABP, particularly in patients with chronic hypertension and diabetes [17]. Medical therapy with beta-blockers and calcium-channel antagonists [8,17], as well as the different levels of barometric pressure utilized during HBOT [8] may account for the differences in the reported proportional rise of ABP. Moreover, clinical predictors for HBOT-induced ABP increase have not been previously described. The purpose of this study was to quantify and examine predictors of ABP changes in patients undergoing HBOT.

## 2. Materials and Methods

### 2.1. Design

This was a retrospective longitudinal cohort study examining the relationship between HBOT and ABP in the elective patients undergoing hyperbaric treatment for the Health Canada approved indications. This study was guided by three research questions. First, what is the temporal relationship between HBOT and ABP? Second, do any of the recorded clinical factors affect changes in ABP following HBOT? Third, are there modifiable clinical predictors that may affect changes in ABP following HBOT? After obtaining institutional Research Ethics Board approval (CAPCR ID: 19-5081.1), medical chart reviews were conducted by study team members.

### 2.2. Subjects

Patients receiving elective HBOT between June 2017 and December 2018 in the Hyperbaric Medicine Unit at the Toronto General Hospital in Toronto, Ontario, Canada, were included in this analysis. Participants were eligible for study inclusion if they met the following criteria: (1) age 18-years or older; (2) scheduled for elective HBOT; (3) Health Canada approved indication requiring at least 20 HBOT sessions.

### 2.3. Hyperbaric Oxygen Therapy (HBOT) Protocol

Standard HBOT protocols were performed for all patients and included administration of 100% oxygen at 2.0 or 2.4 atmospheres absolute (ATA) in one of the three mono-place chambers (Sechrist 3600H and Sechrist 4100H, Sechrist Industries Inc., Anaheim, CA, USA; PAH-S1-3200, Pan-America Hyperbarics Inc., Plano, TX, USA) or through a plastic hood in a multi-place chamber (rectangular Hyperbaric System, Fink Engineering PTY-LTD, Warana, Australia). Standard monitoring included measurements of systolic (SAP), diastolic (DAP), and mean (MAP) ABP as well as heart rate within 1–5 min prior to and after each HBOT session. ABP was measured non-invasively using an upper arm cuff automated sphygmomanometer (Connex VSM 6000, WelchAllyn—Hill-Rom, New York, NY, USA) with the patient in a sitting position (when HBOT was performed in the multi-place chamber) or semi-sitting position on a hyperbaric stretcher (when HBOT was performed in a mono-place chamber). Baseline ABP was classified into four categories based on the clinical practice guidelines by the American College of Cardiology and American Heart Association [18]: (i) Normal: SAP < 120 mmHg and DAP < 80 mmHg, (ii) Elevated: SAP 120–129 mmHg and DAP < 80 mmHg, (iii) Hypertension Stage 1 (stage 1 HTN): SAP 130–139 mmHg or DAP 80–89 mmHg, and (iv) Hypertension Stage 2 (stage 2 HTN): SAP > 140 mmHg or DAP > 90 mmHg.

### 2.4. Outcomes and Extracted Data

The primary outcome was to determine a temporal relationship between ABP and HBOT. SAP, DAP, and MAP were recorded before and after each HBOT session. Demographic variables past medical history and medications were extracted from medical charts. Additional data extracted during the treatment period included HBOT pressure, number of HBOT sessions, and adverse effects associated with HBOT. The secondary outcome was to determine the predictive relationship between demographic and clinical variables and HBOT-related changes in ABP.

### 2.5. Statistical Analysis

Baseline demographic and clinical characteristics were summarized using descriptive statistics examined by Chi-square (or Fisher’s Exact test, where appropriate) and independent sample *t*-tests. Linear mixed effect models were used to determine the predictive relationship between demographic and clinical variables and to estimate mean changes in SAP, DAP, and MAP. The independent variables in the regression analyses were determined a priori and included: age, sex, body mass index, baseline ABP classification as an ordered factor (normotensive, elevated, stage 1 HTN, and stage 2 HTN), use of medication) (i) beta-blocker and/or calcium channel blocker; (ii) angiotensin converting enzyme (ACE) inhibitor, angiotensin receptors blockers (ARB); or (iii) combination of (i) and (ii)), treatment pressure (2.0 ATA or 2.4 ATA); and clinical history of hypertension, lung disease, kidney failure, and diabetes (type I or type II) as fixed effects. Individual subjects were included as random effects. In the aforementioned model, clinical history of hypertension and baseline ABP classification were found to be significant predictors, thus we further examined the effect of the baseline ABP classification on the HBOT-related ABP change by including a time point × baseline ABP classification interaction. Furthermore, to examine the relationship in the pre- and post-HBOT change across treatment sessions, absolute changes in ABP with the interaction of the treatment session number were added to the aforementioned model. Adjusted mean blood pressure for the overall cohort, differences between pre- and post-HBOT, and between ABP classification differences were provided under the maximum likelihood estimation. Pairwise comparisons were adjusted with Tukey’s honestly significant difference (HSD). All analyses were conducted using R version 3.6.1 (R Foundation for Statistical Computing, Vienna, Austria) and alpha was set to 0.05.

## 3. Results

### 3.1. Participant Characteristics

Table 1 summarizes the sample and HBOT characteristics. During the data collection period, 108 patients were included in the study and underwent HBOT, collectively completing 3291 HBOT sessions. The mean age of the patients was 59.95 ± 14.90 years and 56 (52%) were male. An average of 30 ± 11 sessions were completed by patients and the vast majority (78.9%) were delivered at 2.4 ATA. At baseline, 41 (38%), 14 (13%), 39 (36%), and 14 (13%) patients had ABP classifications categorized as normotensive, elevated, hypertension stage 1, and hypertension stage 2, respectively.

### 3.2. Acute Effect of HBOT on Arterial Blood Pressure (ABP)

Changes in ABP from pre to post-HBOT are summarized in Table 2. MAP, SAP, and DAP increased by 6.6 mmHg (95% CI: 6.0, 7.2), 11.3 mmHg (95% CI: 10.3, 12.3), and 4.3 mmHg (95% CI: 3.7, 4.8), respectively. Among the 3291 HBOT sessions analyzed, an increase in SAP and DAP that reached the definition of hypertensive crisis (SAP >180 or DAP >120 mmHg) following an HBOT session that occurred in 151 sessions (4.6%) and 3 sessions (0.09%), respectively. Pre-session ABP for the vast majority was categorized as stage 1 HTN (28 sessions) or stage 2 HTN (94 sessions). On these occasions where hypertensive crisis occurred, all patients were asymptomatic and the ABP returned in the normal range within few minutes without interventions.

### 3.3. Cumulative Effect of HBOT on ABP

**Figure 1:**
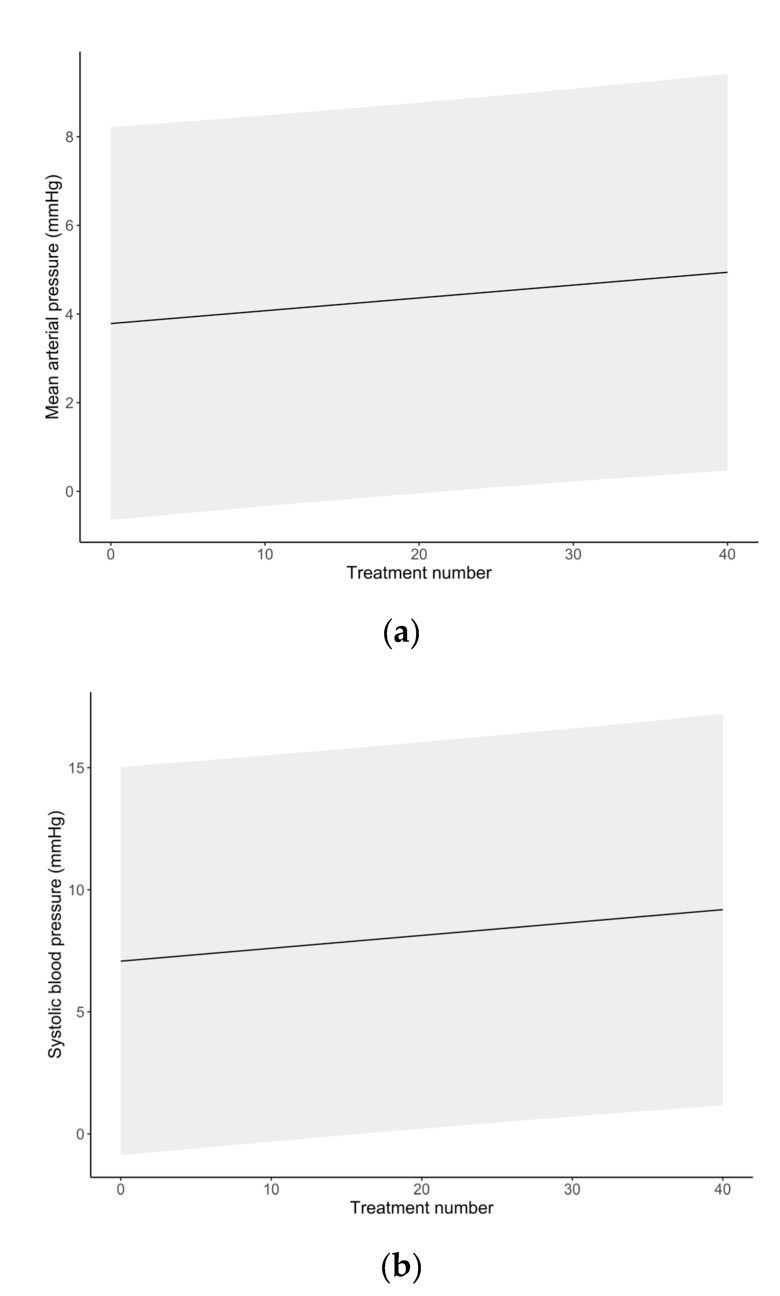

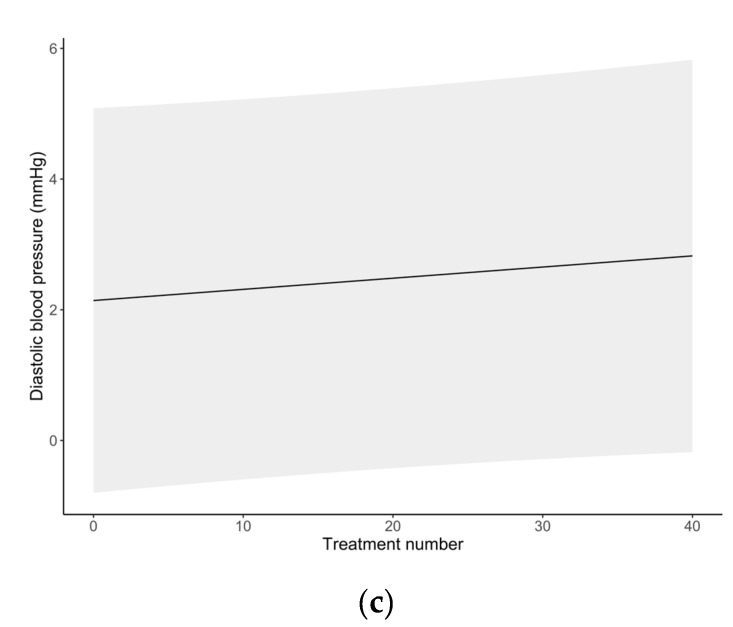
Temporal changes of arterial blood pressure over the total number of HBOT sessions. (**a**) Mean arterial pressure; (**b**) systolic arterial pressure; (**c**) diastolic arterial pressure. Mean post-session ABP values at each session are represented by a point. The shaded area represents the 95% confidence interval.

**Figure 2:**
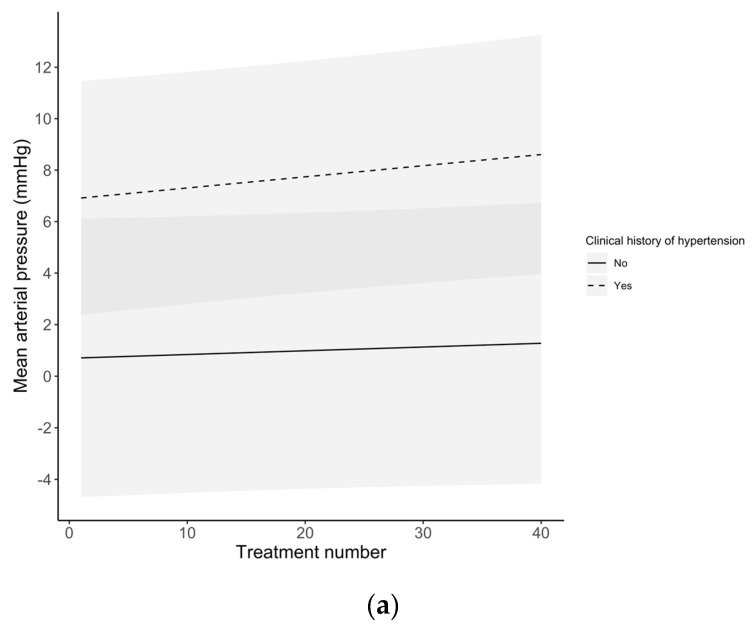

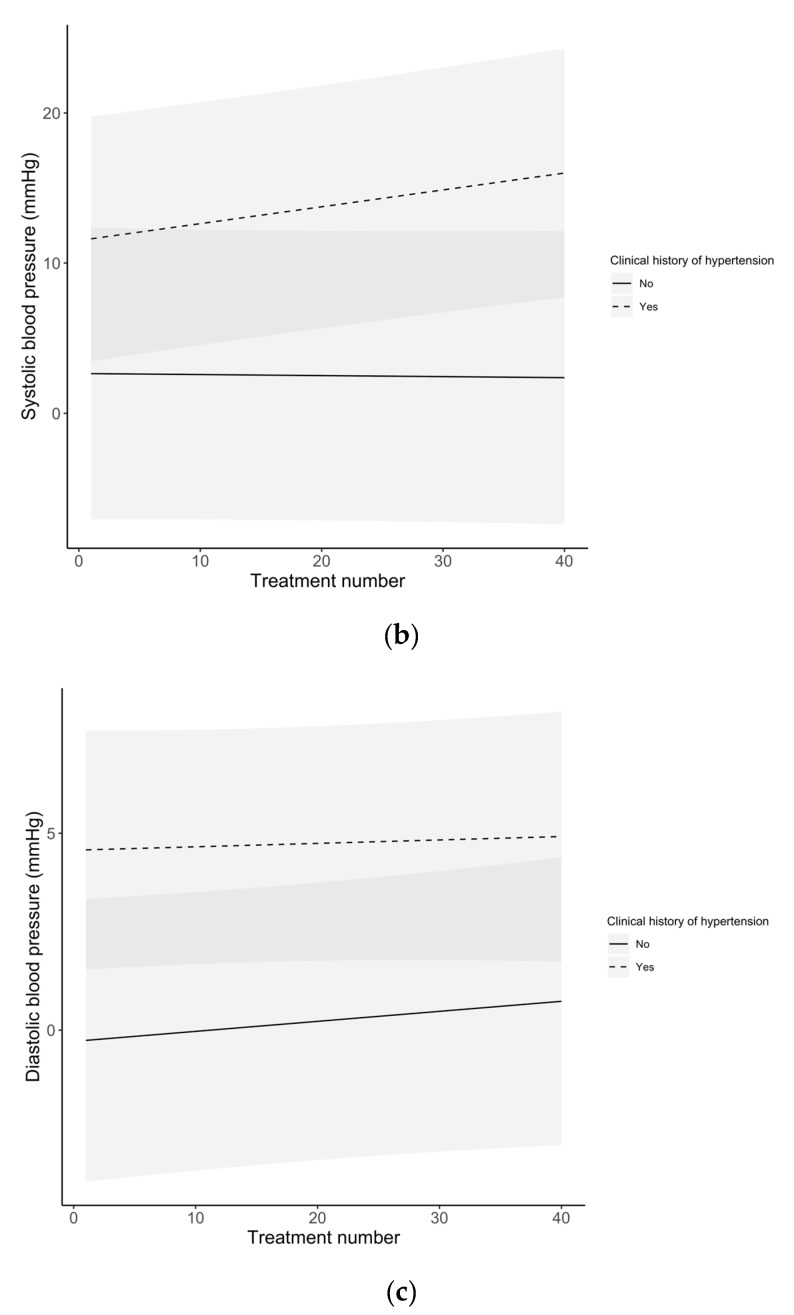
Temporal changes of arterial blood pressure over the total number of HBOT sessions stratified by the history of hypertension. (**a**) Mean arterial pressure; (**b**) systolic arterial pressure; (**c**) diastolic arterial pressure. The shaded area represents the 95% confidence interval.

Differences in ABP changes with each session over the entire course of treatment are summarized in Figure 1. The SAP slightly increased after each treatment, from 7.1 ± 4.1 mmHg after the first session to 9.2 ± 4.1 mmHg after the 40th session. Table 3 summarizes the mean change in ABP measurements after each HBOT session stratified by clinical history of hypertension (derived from medical records). Figure 2 depicts the change in ABP measures with each session across the entire treatment. Across treatment sessions, hypertensive patients exhibited greater change in ABP measures compared to normotensive patients.

### 3.4. Predictors of HBOT-Related Changes in ABP

The model results describing the predictors of HBOT-related changes in ABP are presented in Table 4. Orthogonal polynomial contrast demonstrated a linear relationship between ABP classification at baseline with higher post-HBOT ABP (MAP, β = 14.8, *p* ≤ 0.001; SAP: β = 26.4, *p* ≤ 0.001; DAP: β = 9.0, *p* ≤ 0.001). History of HTN significantly predicted change in MAP (β = 14.8, *p* ≤ 0.001), but not SAP or DAP (SAP: β = 6.1, *p* = 0.063; DAP: β = 3.0, *p* = 0.095).

**Figure 3:**
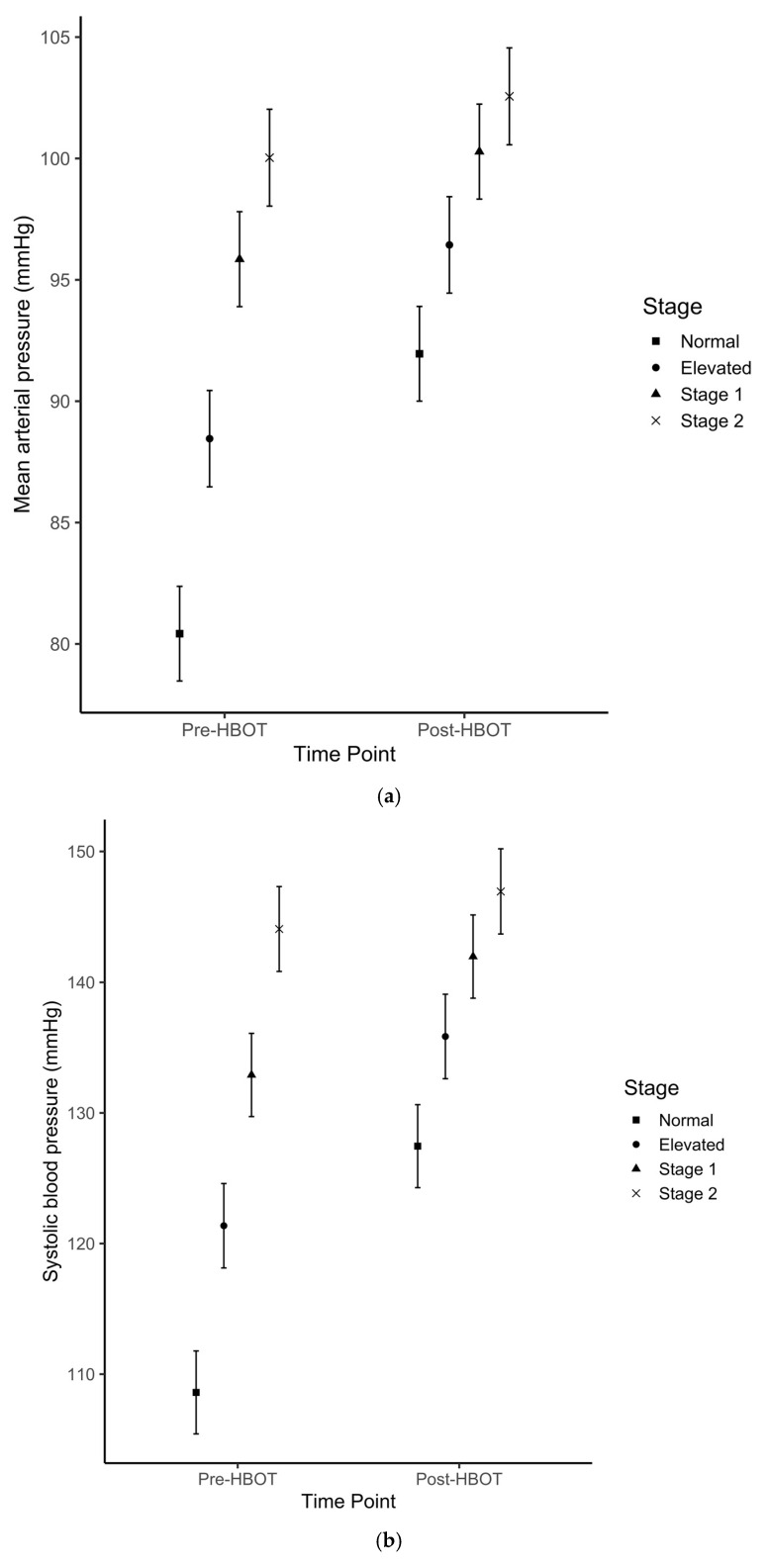

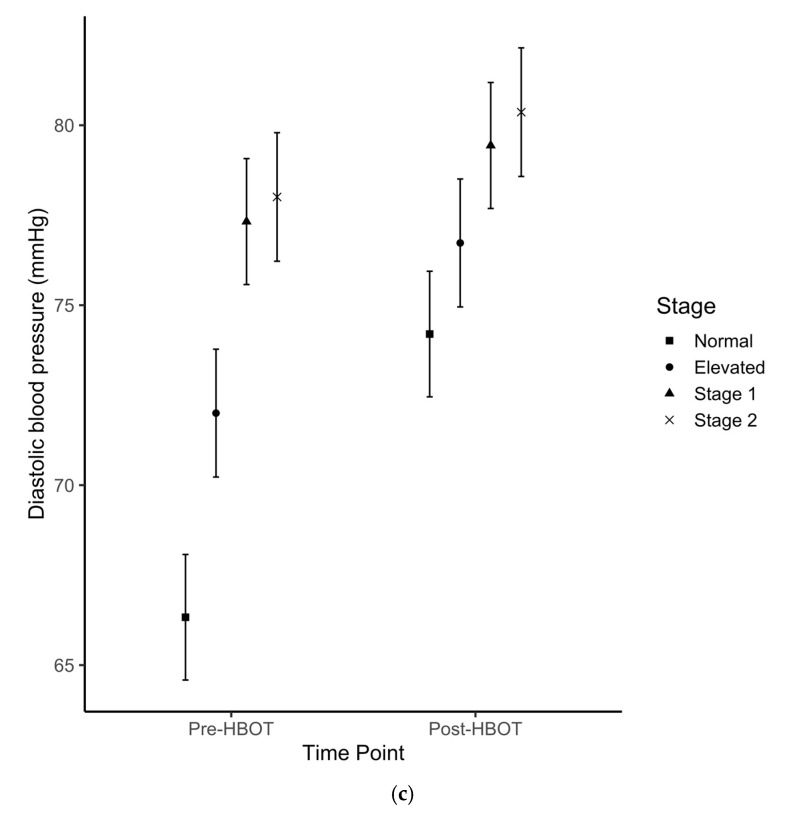
Estimated means ± SE for (**a**) mean-, (**b**) systolic- and (**c**) diastolic arterial pressures before and after hyperbaric oxygen treatment sessions across four categories of baseline arterial blood pressure measurements. Baseline arterial blood pressure classification: Normotensive; Elevated; Hypertension Stage 1; Hypertension Stage 2.

MAP, SAP and DAP changes from pre-HBOT to post-HBOT stratified by baseline (pre-session measurement) ABP classification ranged from 2.1 to 18.9 mmHg (*p* < 0.01) and are presented in Table 5. The data trends indicate that there was a decreasing ABP change (from pre- to post-HBOT) by classification from normotensive to stage 2 HTN (Figure 3). Interaction analyses evaluating the contrasts between the ABP changes across different baseline ABP classifications (e.g., difference in ABP rise between ABP classified as *normal* before HBOT compared to ABP classified as *elevated* before HBOT) is shown in Table 6 and indicated that the greatest change difference occurred between normotensive and stage 2 patients across all measures (MAP, ∆-9.0 mmHg, 95% CI: –12.2, –5.8; SAP: ∆-16.0 mmHg, 95% CI: –21.2, –10.8; DAP: ∆-5.5 mmHg, 95% CI: –8.4, –2.6).

## 4. Discussion

The current study demonstrated a significant absolute rise in ABP after HBOT as compared to before HBOT. The change in ABP was shown to be most pronounced for SAP with a 9.8% increase, followed by MAP with an 8.3% increase, and DAP with a 6.7% increase (Table 2). Several hypotheses exist in recent literature as to how HBOT may account for the observed elevated ABP after hyperbaric oxygen treatment. It is suspected that such hemodynamic changes observed during and after HBOT can be ascribed to a protective vasoconstriction response [6]; however, the magnitude of this response is not well described, and conflicting scientific evidence is found in the literature. In a recent report, Heyboer et al. (2017) [8] evaluated 155 elective patients, for a total of 3147 HBOT sessions, and showed an increase in ABP after treatment in the range of 4–7 mmHg [8]. These findings were in accordance with several additional studies that found no significant changes in ABP following HBOT [15,19,20]. In contrast, Al-Waili et al. [17] showed a significant rise in ABP ranging from 11–12% in a cohort of 49 elective patients receiving at least 15 hyperbaric oxygen treatments. In agreement, our study confirmed that the HBOT-induced rise in ABP, particularly SAP, was statistically significant. Furthermore, this change in ABP was modest in patients with no history of hypertension, but became statistically significant and clinically relevant in patients with a history of hypertension (Table 3) in agreement with Shenouda et al. [21]. In addition to the relationship between a history of hypertension and an increased risk of ABP rise following HBOT, a multivariate regression analysis revealed the importance of patients’ baseline ABP that was measured immediately prior to HBOT. Baseline ABP predicted the extent of ABP increase after HBOT (Table 4 and Table 5). A paradoxical effect of larger median changes in ABP categories was observed at lower ABP baseline measurements. It should be also noted that while the group with normal ABP experienced the largest increase in SAP following HBOT, these changes were not clinically relevant. In contrast, the changes observed in both hypertension groups—while modest in comparison to the normative group—resulted in clinically relevant increases in SAP after HBOT [18]. Lower baseline ABP presumably predisposed patients to a greater increase in ABP after HBOT, thus future work is warranted to determine reproducibility of this effect. Interestingly, in contrast to Heyboer et al. [8], the current study showed an exponential increase in ABP after each HBOT treatment (Figure 1). This trend was more evident between patients with a history of hypertension compared to normotensive patients (Figure 2); it is likely that the vasoconstrictive response becomes more pronounced after treatment, and it may either represent an improved arteriolar smooth muscle activity or a lack of vasorelaxation. While it has been described that repetitive HBOT improves the vasorelaxation [22,23,24], increased vascular stiffness might play a role in impairing this response in patients with a history of hypertension.

Based on these results, patients may benefit from risk stratification prior to HBOT treatment using pre-HBOT ABP with SAP >130 mmHg and/or DAP >80 mmHg as risk factors. It should be noted that none of the patients experienced serious adverse effects during or after HBOT treatment session, and no patient developed hypertensive crises requiring emergency treatment. The most plausible explanation is that patients with SAP >180 or DAP >100 mmHg measured before HBOT are not treated in our institution until their APB is stabilized. All patients who developed transient rise in ABP post-HBOT are referred to their family doctor for optimization of their chronic hypertension management. Our study indicates that all patients undergoing HBOT should have routine ABP measurements before and after the treatments in order to identify new onset and worsening hypertension. The information that hypertension can worsen during the HBOT course justifies the APB optimization to avoid end-organ damage.

The current study has several limitations. First, using a retrospective cohort study design may have increased the risk for selection bias [25]. By design, this study used health records that have already been collected and thus not all pertinent risk factors were likely identified and recorded. Unlike a prospective study, this study involved different healthcare professionals delivering patient care, and thus the measurement of outcomes throughout the database would be less accurate and consistent. Second, despite the overall adequate sample size, stratification of variables (e.g., ATA, medications) led to small subgroup populations. Third, the optimal timeframe for measuring BP following HBOT is not known, and thus, the measurements taken at 1–5 min following the end of each session may not provide the most accurate results. Given this limitation, a real-time ‘inside-the-chamber’ BP monitoring approach—nowadays unavailable for elective patients—and measurements taken at multiple time-points immediately following the end of an HBOT session are recommended for future studies. Finally, HBOT for elective patients with pre-treatment SAP >180 or DAP >100 mmHg are usually postponed or cancelled in our institution until APB is stabilized and, therefore, ABP response of these patients to HBOT was not measured and reported in our study.

## 5. Conclusions

The current study demonstrates that an absolute rise in ABP, particularly SAP, occurs as a result of HBOT. This change in ABP following HBOT is modest in patients with no history of hypertension but becomes clinically relevant in patients with a history of hypertension. Furthermore, the importance of patients’ baseline pre-HBOT BP emerged, with lower baseline ABP predisposing patients to a greater increase in ABP post-HBOT, but also higher baseline ABP as a risk for clinically relevant ABP change after HBOT. Given these findings, pre-session ABP should be used clinically as an indicator for strict monitoring of BP during HBOT, and future studies are recommended to explore evidence-based pre-session ABP thresholds in order to optimize the ABP before starting an HBO treatment.

## Figures and Tables

**Table 1:** Baseline demographics, comorbidities, and medications of the total cohort of 108 patients.

	Total (*n* = 108)	Female (*n* = 52)	Male (*n* = 56)
Age (years)	59.9 ± 15	58.71 (15.94)	61.11 (13.90)
Body Mass Index (kg/m^2^)	26.9 ± 11	27.32 (14.21)	26.38 (5.67)
Female	52 (48)		
**Comorbidities**			
History of hypertension	53 (49)	27 (51.9)	26 (46.4)
Baseline arterial blood pressure classification:			
*Normotensive*	41 (38)	25 (48.1)	15 (27.3)
*Elevated*	14 (13)	7 (13.5)	7 (12.7)
*Hypertension Stage 1*	39 (36)	13 (25.0)	26 (47.3)
*Hypertension Stage 2*	14 (13)	7 (13.5)	7 (12.7)
Coronary artery disease	15 (14)	4 (7.7)	11 (19.6)
Congestive heart failure	11 (10)	5 (9.6)	6 (10.7)
Left ventricular hypertrophy	3 (3)	0 (0.0)	3 (5.4)
Heart valvular disease	11 (10)	4 (7.7)	7 (12.5)
Diastolic dysfunction	1(1)	1 (1.9)	0 (0.0)
Atrial fibrillation	13 (12)	8 (15.4)	5 (8.9)
Peripheral vascular disease	23 (21)	9 (17.3)	14 (25.0)
Diabetes mellitus:			
Type 1	5(5)	3 (5.8)	2 (3.6)
Type 2	24 (22)	11 (21.2)	13 (23.2)
Chronic obstructive pulmonary disease	21 (19)	16 (30.8)	5 (8.9)
Restrictive lung disease	2 (2)	1 (1.9)	1 (1.8)
Smoking status:			
Never	56 (51)	1 (1.9)	1 (1.8)
Current	23 (21)	29 (55.8)	26 (46.4)
Past	30 (28)	7 (13.5)	16 (28.6)
Renal insufficiency Dialysis	22 (20) 12 (11)	1 (21.2) 4 (7.7)	11 (19.6) 8 (14.3)
**Medications**			
ACEi/ARBs	26 (24)	13 (25.0)	13 (23.2)
*β*-blockers	24 (22)	12 (23.1)	12 (21.4)
Calcium channel blockers	28 (26)	15 (28.8)	13 (23.2)
Diuretics	14 (13)	7 (13.5)	7 (12.5)
Vasodilators	9 (8)	5 (9.6)	4 (7.1)
**HBOT** Pressure (2.4 ATA)	86 (79)	39 (75.0)	46 (82.1)

Data expressed as mean ± standard deviation (SD) or frequency (%). HBOT, hyperbaric oxygen therapy; ATA, atmosphere absolute; ACEi, Angiotensin-converting-enzyme inhibitors; ARBs, Angiotensin II receptor blockers.

**Table 2:** Temporal dynamics in arterial blood pressure measurements before and after each HBOT sessions.

ABP Measure	Pre-HBOT (mmHg)	Post-HBOT (mmHg)	∆ (95% CI)
Mean arterial pressure	91.2 ± 1.9	97.8 ± 1.9	6.6 (6.0, 7.2) *
Systolic arterial pressure	127.0 ± 3.1	138.0 ± 3.1	11.3 (10.3, 12.3) *
Diastolic arterial pressure	73.4 ± 1.7	77.7 ± 1.7	4.3 (3.7, 4.8) *

Data expressed as mean ± standard error (SE); * *p* < 0.001.ABP, arterial blood pressure; HBOT, hyperbaric oxygen therapy.

**Table 3:** Temporal dynamics in arterial blood pressure measurements before and after HBOT sessions (entire HBOT course) in patients with or without the history of hypertension.

ABP Measure	Clinical History of Hypertension	Estimated Mean Difference after HBOT
Mean arterial pressure	Normotensive	1.0 ± 2.7
	Hypertensive	7.7 ± 2.3
Systolic arterial pressure	Normotensive	2.5 ± 4.9
	Hypertensive	13.6 ± 4.1
Diastolic arterial pressure	Normotensive	0.2 ± 1.8
	Hypertensive	4.7 ± 1.5

ABP, arterial blood pressure; HBOT, hyperbaric oxygen therapy.

**Table 4:** Linear mixed effect model evaluating patients’ demographic and clinical factors pertinent to arterial blood pressure after HBOT sessions.

Fixed Effect	Mean Arterial Pressure			Systolic Arterial Pressure			Diastolic Arterial Pressure		
	β	SE	*p*	β	SE	*p*	β	SE	*p*
Intercept	88.4	4.4	<0.001	105.7	7.2	<0.001	79.7	4.0	<0.001
Measure time point (post-HBOT)	6.6	0.3	<0.001	11.3	0.5	<0.001	4.3	0.3	<0.001
Blood pressure classification	14.8	0.5	<0.001	26.4	0.8	<0.001	9.0	0.4	<0.001
Age	0.1	0.1	0.284	0.3	0.1	0.004	−0.1	0.1	0.345
Medications									
β-blockers	−1.4	2.4	0.555	−2.0	3.9	0.603	−1.1	2.2	0.605
ACEi/ARBs	0.8	5.9	0.891	−5.6	9.6	0.558	4.0	5.3	0.446
Combination	−3.5	2.5	0.169	−4.1	4.1	0.325	−3.2	2.2	0.158
History of hypertension (yes)	4.0	2.0	0.046	6.1	3.2	0.063	3.0	1.8	0.095
History of lung disease (yes)	−1.4	1.9	0.474	−0.9	3.2	0.778	−1.7	1.7	0.345
Body Mass Index	−0.1	0.1	0.185	−0.2	0.1	0.228	−0.1	0.1	0.259
ATA >2.0 (2.4)	−0.1	1.8	0.972	4.0	3.0	0.180	−2.1	1.6	0.201
Male gender	2.5	1.6	0.130	3.9	2.7	0.153	1.8	1.5	0.212
Diabetes mellitus									
Type I	−1.4	3.8	0.716	0.3	6.1	0.965	−2.2	3.4	0.515
Type II	1.3	2.1	0.534	6.7	3.4	0.056	−1.4	1.9	0.471
**Random effects**	**Variance**	**SD**		**Variance**	**SD**		**Variance**	**SD**	
Participants	25.9	5.1		68.9	8.3		20.7	4.6	
Residual	74.8	8.6		196.8	14.0		61.5	7.8	
**Marginal/Conditional R^2^**	0.42/0.57			0.47/0.61			0.27/0.46		

Data expressed as degree of change in arterial blood pressure (β, mmHg); SE, standard error; *p* < 0.05 was considered statistically significant. HBOT, hyperbaric oxygen therapy. ATA, atmosphere absolute; ACEI, Angiotensin-converting-enzyme inhibitors; ARBs, Angiotensin II receptor blockers.

**Table 5:** Arterial blood pressure changes before and after HBOT in patients stratified by hypertension classification based on baseline arterial blood pressure.

ABP Measure	Class	Pre-HBOT	Post-HBOT	∆ (95% CI)
Mean arterial pressure, mmHg	Normotensive	80.4 ± 1.9	92.0 ± 2.0	11.5 (10.6, 12.4) *
	Elevated	88.5 ± 2.0	96.4 ± 2.0	8.0 (6.5, 9.4) *
	Stage I HTN	95.9 ± 2.0	100.3 ± 2.0	4.4 (3.3, 5.5) *
	Stage 2 HTN	100.0 ± 2.0	102.6 ± 2.0	2.5 (1.1, 3.9) *
Systolic arterial pressure, mmHg	Normotensive	108.6 ± 3.2	127.5 ± 3.2	18.9 (17.4, 20.3) *
	Elevated	121.4 ± 3.2	135.9 ± 3.2	14.5 (12.1, 16.8) *
	Stage I HTN	132.9 ± 3.2	142.0 ± 3.2	9.1 (7.3, 10.8) *
	Stage 2 HTN	144.1 ± 3.2	147.0 ± 3.3	2.9 (0.6, 5.2) **
Diastolic arterial pressure, mmHg	Normotensive	66.3 ± 1.7	74.2 ± 1.7	7.9 (7.0, 8.7) *
	Elevated	72.0 ± 1.8	76.7 ± 1.8	4.7 (3.4, 6.0) *
	Stage I HTN	77.3 ± 1.7	79.4 ± 1.8	2.1 (1.1, 3.1) *
	Stage 2 HTN	78.0 ± 1.8	80.4 ± 1.8	2.4 (1.1, 3.6) *

Data expressed as number ± standard error (SE). * *p* < 0.001, ** *p* < 0.05. ABP, arterial blood pressure; HBOT, hyperbaric oxygen therapy; HTN, hypertension.

**Table 6:** Contrasts between changes in arterial blood pressure before and after HBOT, across different baseline blood pressure categories.

Variable	Categories	∆ (95% CI)	*p*-Value
Mean arterial pressure	Elevated—normotensive	−3.5 (−6.9, −0.3)	0.015
	Stage 1—normotensive	−7.1 (−9.8, −4.4)	<0.001
	Stage 2—normotensive	−9.0 (−12.2, −5.8)	<0.001
	Stage 1—elevated	−3.6 (−7.0, −0.1)	0.033
	Stage 2—elevated	−5.5 (−9.3, −1.6)	<0.001
	Stage 2—Stage 1	−1.9 (−5.3, 1.5)	0.952
Systolic arterial pressure	Elevated—normotensive	−4.4 (−9.6, 0.9)	0.303
	Stage 1—normotensive	−9.8 (−14.2, −5.4)	<0.001
	Stage 2—normotensive	−16.0 (−21.2, −10.8)	<0.001
	Stage 1—elevated	−5.4 (−11.0, 0.16)	0.071
	Stage 2—elevated	−11.6 (−17.9, −5.4)	<0.001
	Stage 2—Stage 1	−6.2 (−11.7, −0.7)	0.008
Diastolic arterial pressure	Elevated—normotensive	−3.1 (−6.1, −0.2)	0.021
	Stage 1—normotensive	−5.7 (−8.2, −3.3)	<0.001
	Stage 2—normotensive	−5.5 (−8.4, −2.6)	<0.001
	Stage 1—elevated	−2.6 (−5.7, 0.5)	0.279
	Stage 2—elevated	−2.5 (−5.9, 1.1)	0.730
	Stage 2—Stage 1	0.2 (−2.8, 3.3)	1.00

## Abbreviations

ABP	arterial blood pressure
ACEi	Angiotensin-converting-enzyme inhibitors
ARBs	Angiotensin II receptor blockers
ATA	atmospheres absolute
DAP	diastolic blood pressure
HBOT	hyperbaric oxygen therapy
HSD (Tukey’s HSD)	Tukey’s honestly significant difference
HTN	hypertension
MAP	mean arterial pressure
SAP	systolic blood pressure

## References

[1] Moon R.E. (2019). Hyperbaric Oxygen Therapy Indications.

[2] Jain K.K., Jain K.K. (2017). Indications, Contraindications, and Complications of HBO Therapy. Textbook of Hyperbaric Medicine.

[3] Shupak A., Gilbey P. (2008). Effects of Pressure. Physiology and Medicine of Hyperbaric Oxygen Therapy.

[4] Heyboer M., Sharma D., Santiago W., McCulloch N. (2017). Hyperbaric Oxygen Therapy: Side Effects Defined and Quantified. Adv. Wound Care.

[5] Heyboer M., Wojcik S.M., Grant W.D., Chambers P., Jennings S., Adcock P. (2014). Middle ear barotrauma in hyperbaric oxygen therapy. Undersea Hyperb. Med..

[6] Mathieu D., Favory R., Collet F., Linke J.-C., Wattel F. (2006). Physiologic Effects of Hyperbaric Oxygen on Hemodynamics and Microcirculation. Handbook on Hyperbaric Medicine.

[7] Nakada T., Koike H., Katayama T., Watanabe H., Yamori Y. (1984). Increased adrenal epinephrine and norepinephrine in spontaneously hypertensive rats treated with hyperbaric oxygen. Hinyokika Kiyo..

[8] Heyboer Iii M., Wojcik S., Smith G., Santiago W. (2017). Effect of hyperbaric oxygen therapy on blood pressure in patients undergoing treatment. Undersea Hyperb. Med..

[9] Demchenko I.T., Zhilyaev S.Y., Moskvin A.N., Krivchenko A.I., Piantadosi C.A., Allen B.W. (2013). Baroreflex-mediated cardiovascular responses to hyperbaric oxygen. J. Appl. Physiol..

[10] Bove A.A. (2008). Cardiovascular Aspects of Hyperbaric Oxygen Therapy. Physiology and Medicine of Hyperbaric Oxygen Therapy.

[11] Whitehorn W.V., Edelmann A., Hitchcock F.A. (1946). The Cardiovascular Responses to the Breathing of 100 per Cent Oxygen at Normal Barometric Pressure. Am. J. Physiol. Leg. Content.

[12] Alveryd A.L.V., Brody S.A.M. (1948). Cardiovascular and Respiratory Changes in Man during Oxygen Breathing. Acta Physiol. Scand..

[13] Mulkey D.K., Henderson R.A., Putnam R.W., Dean J.B. (2003). Pressure (≤4 ATA) increases membrane conductance and firing rate in the rat solitary complex. J. Appl. Physiol..

[14] Linnarsson D., Östlund A., Lind F., Hesser C.M. (1999). Hyperbaric bradycardia and hypoventilation in exercising men: Effects of ambient pressure and breathing gas. J. Appl. Physiol..

[15] Whalen R.E., Saltzman H.A., Holloway D.H., McIntosh H.D., Sieker H.O., Brown I.W. (1965). Cardiovascular and blood gas responses to hyperbaric oxygenation. Am. J. Cardiol..

[16] Weaver L.K., Howe S., Snow G.L., Deru K. (2009). Arterial and pulmonary arterial hemodynamics and oxygen delivery/extraction in normal humans exposed to hyperbaric air and oxygen. J. Appl. Physiol..

[17] Al-Waili N.S., Butler G.J., Beale J., Abdullah M.S., Finkelstein M., Merrow M., Rivera R., Petrillo R., Carrey Z., Lee B. (2006). Influences of Hyperbaric Oxygen on Blood Pressure, Heart Rate and Blood Glucose Levels in Patients with Diabetes Mellitus and Hypertension. Arch. Med. Res..

[18] Whelton P.K., Carey R.M., Aronow W.S., Casey D.E., Collins K.J., Dennison Himmelfarb C., DePalma S.M., Gidding S., Jamerson K.A., Jones D.W. (2018). 2017 ACC/AHA/AAPA/ABC/ACPM/AGS/APhA/ASH/ASPC/NMA/PCNA Guideline for the Prevention, Detection, Evaluation, and Management of High Blood Pressure in Adults: A Report of the American College of Cardiology/American Heart Association Task Force on Clinical Practice Guidelines. J. Am. Coll. Cardiol..

[19] McMahon T.J., Moon R.E., Luschinger B.P., Carraway M.S., Stone A.E., Stolp B.W., Gow A.J., Pawloski J.R., Watke P., Singel D.J. (2002). Nitric oxide in the human respiratory cycle. Nat. Med..

[20] Kenmure A.C., Murdoch W.R., Hutton I., Cameron A.J. (1972). Hemodynamic effects of oxygen at 1 and 2 Ata pressure in healthy subjects. J. Appl. Physiol..

[21] Shenouda M.B.M., Lakdawala S., Sung J., Warhul Z., Bosco G., Camporesi E.M. (2020). Quality Review for Hypertensive Patients Receiving HBO2. Proceedings of the UHMS Annual Scientific Meeting 2020, Virtual Meeting.

[22] Manojlovic D., Stupin A., Mihaljevic Z., Matic A., Lenasi H., Drenjancevic I. (2019). Hyperbaric oxygenation affects acetylcholine-induced relaxation in female diabetic rats. Undersea Hyperb. Med..

[23] Mihaljevic Z., Matic A., Stupin A., Rasic L., Jukic I., Drenjancevic I. (2018). Acute Hyperbaric Oxygenation, Contrary to Intermittent Hyperbaric Oxygenation, Adversely Affects Vasorelaxation in Healthy Sprague-Dawley Rats due to Increased Oxidative Stress. Oxid. Med. Cell Longev..

[24] Hink J., Thom S.R., Simonsen U., Rubin I., Jansen E. (2006). Vascular reactivity and endothelial NOS activity in rat thoracic aorta during and after hyperbaric oxygen exposure. Am. J. Physiol. Heart Circ. Physiol..

[25] Sedgwick P. (2014). Retrospective cohort studies: Advantages and disadvantages. BMJ Br. Med. J..

